# Translational Control through eIF2alpha Phosphorylation during the *Leishmania* Differentiation Process

**DOI:** 10.1371/journal.pone.0035085

**Published:** 2012-05-31

**Authors:** Serge Cloutier, Maxime Laverdière, Marie-Noelle Chou, Nathalie Boilard, Conan Chow, Barbara Papadopoulou

**Affiliations:** Infectious Disease Research Centre, CHUL Research Centre and Department of Microbiology and Immunology, Faculty of Medicine, Laval University, Quebec, Canada; University of Georgia, United States of America

## Abstract

The parasitic protozoan *Leishmania* alternates between an invertebrate and a mammalian host. Upon their entry to mammalian macrophages, *Leishmania* promastigotes differentiate into amastigote forms within the harsh environment of the phagolysosomal compartment. Here, we provide evidence for the importance of translational control during the *Leishmania* differentiation process. We find that exposure of promastigotes to a combined elevated temperature and acidic pH stress, a key signal triggering amastigote differentiation, leads to a marked decrease in global translation initiation, which is associated with eIF2α phosphorylation. Interestingly, we show that amastigotes adapted to grow in a cell-free medium exhibit lower levels of protein synthesis in comparison to promastigotes, suggesting that amastigotes have to enter a slow growth state to adapt to the stressful conditions encountered inside macrophages. Reconversion of amastigotes back to promastigote growth results in upregulation of global translation and a decrease in eIF2α phosphorylation. In addition, we show that while general translation is reduced during amastigote differentiation, translation of amastigote-specific transcripts such as *A2* is preferentially upregulated. We find that *A2* developmental gene regulation is triggered by temperature changes in the environment and that occurs mainly at the level of translation. Upon elevated temperature, the *A2* transcript is stabilized through its association with polyribosomes leading to high levels of translation. When temperature decreases during amastigote to promastigote differentiation, the *A2* transcript is not longer associated with translating polyribosomes and is being gradually degraded. Overall, these findings contribute to our better understanding of the adaptive responses of *Leishmania* to stress during its development and highlight the importance of translational control in promastigote to amastigote differentiation and vice-versa.

## Introduction

*Leishmania* spp. are the causative agents of leishmaniasis, the collective name for a number of important parasitic diseases affecting over 12 million people in 88 countries worldwide [1]. These parasites exist in two major developmental stages; free-living flagellated promastigotes in the midgut of the sand fly vector and non-motile amastigotes that can replicate in the phagolysosome of mammalian macrophages. During its intracellular development, *Leishmania* undergoes marked morphological and biochemical transformations that are essential for its adaptation and survival to the rapidly changing environments in the macrophage [2], [3], [4], [5]. These changes implicate dynamic alterations in the regulation of gene expression, mostly at the posttranscriptional level (reviewed in [6], [7]).

Environmental stimuli like elevated temperature and low pH have been shown to be crucial in triggering *Leishmania* promastigote to amastigote differentiation *in vitro* [8], [9], [10], [11]. Genome-wide transcriptomics and proteomics studies have shown that during amastigote differentiation, 3%–9% of genes in several *Leishmania* species are regulated at the level of individual mRNAs and up to 12%–18% are modulated at the protein level [12], [13], [14], [15], [16], [17], [18]. Posttranslational modifications (PTMs) are also likely to play a key role in the parasite’s development and in response to stress. For example, different protein isoforms were depicted by proteomic analyses to be specific to either life stages of the parasite [12] and a large number of PTM sites (e.g. methylation, acetylation, phosphorylation, glycosylation) has been identified during the *Leishmania donovani* promastigote to amastigote differentiation *in vitro* [16]. Moreover, proteomic analyses of affinity-enriched phosphoprotein extracts obtained from *L. donovani* promastigotes and axenic amastigotes revealed important differences in protein phosphorylation profiles across the two major *Leishmania* lifestages [19], [20], [21].

In eukaryotes, one of the best conserved stress response pathway is the phosphorylation of the alpha-subunit of eukaryotic initiation factor-2 (eIF2α) at serine 51 by stress-responsive kinases, resulting in the reduction of global translation [22], [23], [24], [25]. The phosphorylation of eIF2α prevents GDP-GTP exchange on eIF2 by the guanine nucleotide exchange factor eIF2B, thereby inhibiting recycling of the ternary complex that contains the initiator methionine Met-tRNAi [23], [24], [25]. Consequently, global translation initiation is decreased, so the cell can save energy and modulate gene expression in response to stress. There are four known eIF2α kinases in mammalian cells that phosphorylate eIF2α and are activated by different stresses. These include the double-stranded-RNA dependent protein kinase PKR, an interferon-inducible protein that is an important component in the antiviral response; the general control non-derepressible-2 kinase (GCN2), which is activated in response to amino acid starvation; the heme-regulated inhibitor (HRI) whose activity is induced by heme deficiency; and the endoplasmic reticulum (ER)-resident protein kinase PERK which is activated by the accumulation of unfolded proteins in the ER [25], [26]. Recently, we characterized the *Leishmania* PERK eIF2α kinase homolog and addressed its role in the parasite’s differentiation within macrophages [27]. Most importantly, we showed that lack of eIF2α phosphorylation in a PERK dominant negative mutant overexpressing a truncated PERK protein markedly delayed the *Leishmania* amastigote differentiation process [27].

Here, we report that *Leishmania* promastigotes exposed to a combined high temperature and low pH stress, a key signal triggering amastigote differentiation, exhibit a marked reduction in global translation, which coincides with eIF2α phosphorylation. Protein synthesis is also globally downregulated in axenic *in vitro*-generated amastigotes as compared to promastigotes while translation of known amastigote-specific genes is preferentially increased. Attenuation of global translation during amastigote differentiation may be important for allowing the parasite to adapt to the harsh environment of the phagolysosome in its mammalian host.

## Results

### Exposure of *Leishmania* to Environmental Signals Triggering Amastigote Differentiation Leads to a Decrease in Global Translation

**Figure 1:**
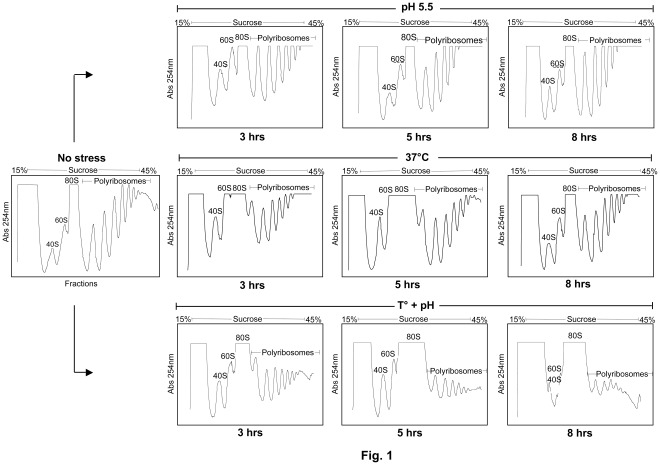
Effect of temperature and pH stress on general translation in *Leishmania* *.* Polysome profile analysis of *L. infantum* promastigotes (no stress; 25°C and pH 7.3) and parasites exposed for different time periods to either elevated temperature (37°C) or low pH (5.5) or to a combined temperature and acidic pH (T°+pH) stress. Both elevated temperature and low pH are major signals triggering amastigote differentiation within the phagolysosome of the host macrophage [8], [10]. *Leishmania* lysates were fractionated by 15% to 45% sucrose density ultracentrifugation and absorbance (Abs) at 254 nm was continuously recorded. The 40S and 60S ribosomal subunits, 80S monosome and polyribosome peaks are indicated. Data displayed here represent one of 3–5 separate experiments.

**Figure 2:**
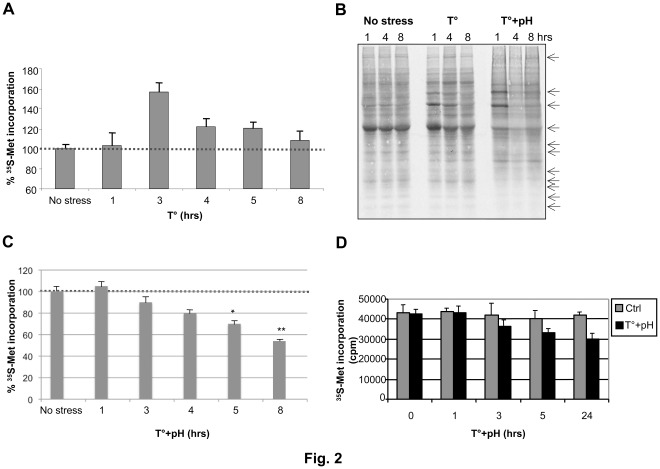
The effect of temperature and acidic pH stress on the *de novo* protein synthesis in *Leishmania*. (A) Levels of [^35^S]-methionine incorporation (as an indicative of general translation rates) in *L. infantum* promastigotes subjected from 1 to 8 hours to either an elevated temperature (37°C) stress (A) or to a combined temperature and low pH (pH 5.5) stress (C). Thirty minutes before protein sample collection, 1 µCi/ml [^35^S]-methionine protein labeling mix was added to the culture media lacking methionine. Protein synthesis was measured as the incorporated radioactivity by a scintillation counter and expressed as cpm. Results are the mean of a minimum of six independent experiments. (B) [^35^S]-Met-labeled proteins resolved on 10% SDS-PAGE. Cells grown at 25°C/pH 7.3 (no stress) or exposed to elevated temperature (37°C) (T°) only or to a combined temperature and acidic pH (pH 5.5) stress for 1, 4 and 8 hours were labelled with [^35^S]-Met for 30 min. An autoradiograph of the SDS-PAGE analysis is shown here. Examples of upregulated or downregulated proteins under stress are indicated by arrows. (D) Pulse-chase assay to assess the stability of *L. infantum* proteins under stress. *L. infantum* promastigotes were incubated in a methionine-deprived medium supplemented with [^35^S]-Met for 1 hour at 25°C and then transferred to a non-radioactive methionine-containing medium at 37°C and pH 5.5 and grown for 24 hours. Aliquots were taken at 1, 3, 5 and 24 hours and the radioactivity (in cpm) was measured by a scintillation counter. A decrease in cpm numbers corresponds to protein degradation under stress. Results are the mean of three independent experiments. Significant differences between the various conditions in (C) are indicated (* p<0.05 and **p<0.01) using one-way ANOVA followed by the Tukey-Kramer test.

Elevated temperature and drop in pH constitute key signals triggering *Leishmania* promastigote to amastigote differentiation within the phagolysosome of mammalian macrophages [4], [8], [10], [28], [29]. To respond to various external stimuli and stresses, the parasite has developed several adaptive strategies allowing dynamic alterations in gene expression, mainly at the post-transcriptional level [11], [13], [14], [15], [16], [19], [30]. To better understand how *Leishmania* adapts and survives within the harsh phagolysosomal compartment, we first investigated the effect of elevated temperature and low pH (differentiation signals) on global translation using sucrose density gradient centrifugation to analyze ribosome profiles and metabolic cell labeling to evaluate protein synthesis rates. *L. infantum* promastigotes grown in RPMI medium at 25°C and pH 7.3 were exposed to either acidic or heat-shock stresses for different time points. Gradual exposure of *L. infantum* promastigotes for 3, 5 and 8 hours to an acidified medium or to a temperature shift from 25°C to 37°C had no significant effect on global translation, as determined by polysome profiling analysis (Figure 1, upper and middle panels). Furthermore, [^35^S]-methionine incorporation into *Leishmania* proteins in parasites grown in methionine-free medium during temperature stress corroborated the polysome profiling data (Figure 2A). Interestingly, 3 hours following temperature stress, a significant increase in [^35^S]-Met labeling (Figure 2A) and the accumulation of high molecular-weight polypeptides (Figure 2B) was observed. These data suggest that translation of specific proteins is increased in response to temperature stress, consistent with previous reports indicating a selective up-regulation of heat-shock proteins and of chaperones to increase cellular survival [31].

We next evaluated the effect of a combined temperature and acidic pH stress on *L. infantum* global translation using polysome profiling analysis and metabolic labeling, as described above. In contrast to the individual temperature or pH stresses, only few hours exposure of *L. infantum* to the combined stress resulted in a marked reduction of general translation, as illustrated by the pronounced decrease in the number and density of polysomes and the concomitant increase in free ribosomal subunits (40S and 60S) and monosomes (80S) (Figure 1, lower panels), which is consistent with reduced rates of translation initiation. A similar effect on translation upon temperature and acidic pH stress was observed with another *Leishmania* species, *L. major* (Figure S1). Moreover, [^35^S]-Met labeling experiments showed more than 20% decrease in *de novo* protein synthesis 5 hours after exposure of the parasite to combined stress and more than 40% decrease 8 hours following the same stress in comparison to the unstressed parasites (Figures 2B and 2C). This marked attenuation in translation was not related to parasite mortality as after 8 hours of exposure to high temperature and low pH, mortality rates of the parasite as assessed by propidium iodide staining were only at 8% in comparison to 2% for the unstressed control (data not shown).

To assess the levels of overall protein degradation under conditions of a combined temperature and pH stress, we carried out pulse-chase assays. Unstressed and stressed parasites were first grown in methionine-free medium to which [^35^S]-Met was added for 1 hour. Then, parasites were chased by replacing [^35^S]-Met by cold methionine in the medium while applying elevated temperature and low pH stresses for 1, 3, 5 and 24 hours. Only 16% of the radiolabeled proteins were degraded after 5 hours of combined stress as estimated by [^35^S]-Met incorporation analysis, but this percentage increased up to 25% following 24 hours of stress whereas less than 2% protein degradation was observed in unstressed parasites treated the same way (Figure 2D). Together, these findings indicate that combined elevated temperature and acidic pH stress represses global mRNA translation and accelerates protein degradation in *Leishmania*.

### *Leishmania* Amastigotes Exhibit Generally Lower Translation Rates than the Highly Replicating Promastigote Forms

**Figure 3:**
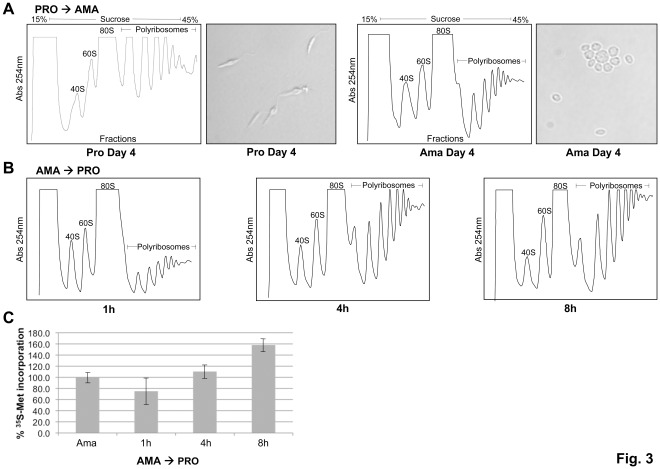
Regulation of general translation throughout the *Leishmania infantum* life cycle. Promastigote to amastigote differentiation (A). Polysome profile analysis of *L. infantum* promastigotes (Pro) and axenic amastigotes (Ama) grown in MAA 20 medium at 37°C and pH 5.5 for 4 days in average (1^st^ passage). *L. infantum* differentiates into amastigote-like forms after ∼4 days of growth under these conditions as illustrated in (A). Amastigote to promastigote differentiation (B). Polysome profile analysis of *L. infantum* axenic amastigotes transferred from the MAA 20 medium at 37°C and pH 5.5 to a MAA medium at 25°C and pH 7.3 for up to 8 hours. Data displayed here represent one of three separate experiments. (C) The effect of *L. infantum* amastigote to promastigote differentiation on global protein synthesis as estimated by the incorporation levels of [^35^S]-Met into the *Leishmania* proteins (as described in Figure 2). Results are the mean of four independent experiments.

Amastigote differentiation can be induced in a host-free medium by culturing stationary promastigotes of most *Leishmania* species in an environment mimicking the phagolysosomal compartment of macrophages (37°C and pH 5.5 in 5% CO_2_) [4], [9], [32], [33], [34], [35]. Based on a number of criteria, including the parasite’s morphology, infectivity, immunochemistry, biochemical properties and gene expression patterns, axenic amastigotes share extensive similarities with macrophage-derived or lesion-derived amastigotes [4], [10], [13], [14], [15], [28], [34], [35], [36] (Figures 3A and S2). Here, we assessed the rates of global translation throughout the parasite’s life cycle. Differentiation of *L. infantum* promastigotes into amastigote-like forms in MAA-20 cell-free medium at 37°C and pH 5.5 for 4 days led to a significant decrease in global translation, as determined by ribosome profile analysis (Figure 3A). Interestingly, adapted axenic amastigotes grown for five consecutive passages in the MAA-20 medium demonstrated lower translation rates in comparison to the highly replicating promastigotes grown for the same number of passages (data not shown). Reconversion of axenic amastigotes back to promastigotes increased global translation (Figure 3B) to levels similar to those observed in promastigotes (Figures 1 and 3A, left panel). In line with the polysome profiling data (Figure 3B), [^35^S]-Met labeling experiments showed that amastigote to promastigote differentiation was associated with higher incorporation of ^35^S-Met into *Leishmania* proteins, which was proportional to the duration of growth under promastigote conditions (Figure 3C), consistent with an increase in protein synthesis under those conditions.

### Decrease in General Translation upon Stress and during *Leishmania* Amastigote Differentiation Correlates with eIF2α Phosphorylation

**Figure 4:**
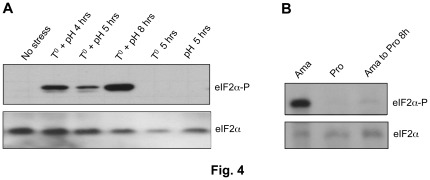
The translation initiation factor 2-alpha subunit (eIF2α) is phosphorylated upon temperature and acidic pH stress and during promastigote to amastigote differentiation. *L. infantum* promastigotes (Pro) (no stress) or promastigotes exposed to elevated temperature (37°C) or to a combined temperature and low pH (pH 5.5) stress for 4, 5 and 8 hours (A) or axenic amastigotes (Ama) and or amastigotes subjected to promastigote differentiation (B) were lysed and whole-cell lysates were used in immunoblots with a rabbit polyclonal anti-eIF2α [pS51] phosphospecific antibody (eIF2α-P) to detect eIF2α phosphorylation. Protein loading was monitored using an anti-*Leishmania* specific eIF2α antibody described in [27].

We next investigated whether attenuation of general translation in *L. infantum* axenic amastigotes is related to eIF2α phosphorylation, a well-documented mechanism to inhibit translation initiation in eukaryotes [25]. Phosphorylated eIF2α inhibits translation initiation, causing dissociation of polysomes and accumulation of monosomes and ribosomal subunits in stressed cells [23]. Western blot analysis using a commercially available phospho-antibody recognizing the phosphorylated form of eIF2α revealed high levels of eIF2α phosphorylation in promastigotes exposed to a combined high temperature and low pH stress for at least 4 hours but not in individually temperature-stressed or pH-stressed parasites or in unstressed promastigotes (Figure 4A). As also reported previously [27], eIF2α phosphorylation is detected in amastigotes (Figure 4B) where general translation is attenuated (Figure 3A) but not or very little in promastigotes undergoing active translation (Figures 1 and 4B). Interestingly, eIF2α phosphorylation drops down to background levels upon reconversion of amastigotes back to promastigotes (Figure 4B). EIF2α phosphorylation coincides with a marked decrease in global translation in parasites exposed to a combined temperature and acidic stress (Figure 1) or during amastigote differentiation *in vitro* (Figure 3A).

### Concomitant Exposure of *Leishmania* to Temperature and Acidic pH Stress Selectively Induces Translation of Developmentally Regulated Genes

Although diverse cellular stress conditions generally repress global protein synthesis, there are examples where translation of specific transcripts is selectively enhanced in response to stressful stimuli [37]. For example, amino-acid starvation enhances translation of *GCN4* [38] in *Saccharomyces cerevisiae* and of *ATF4* [39] and the *cat-1* amino acid transporter [40] in mammalian cells. We therefore investigated the effect of elevated temperature, low pH and amastigote differentiation on the translational efficiency of developmentally regulated transcripts in comparison to housekeeping genes. The constitutively expressed alpha-tubulin gene and the *A2* developmentally regulated gene known to be expressed specifically in *L. donovani* amastigotes [41] were analyzed in this study.

**Figure 5:**
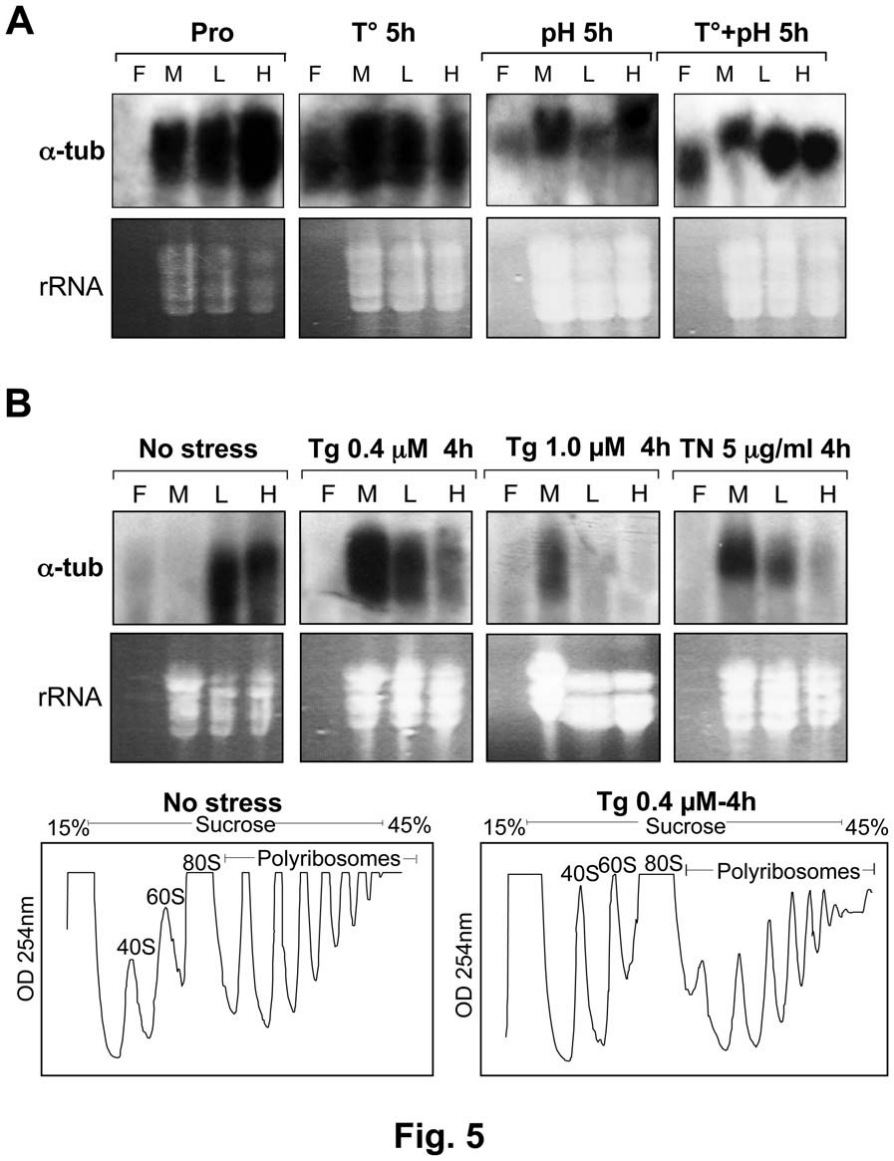
Translation of *Leishmania* housekeeping genes under stress. (A–B) Northern blot hybridization of *L. infantum* RNA samples isolated from sucrose gradient fractions (F: free mRNPs; M: monosomes; L: light polysomes; H: heavy polysomes) to determine the association of the constitutively expressed alpha-tubulin mRNA with translating polysomes in the absence of stress (no stress) or under temperature (T°) or acidic pH stresses (A) and or in the presence of the endoplasmic reticulum (ER)-inducing agents thapsigargin (Tg) or tunicamycin (TN) (B, upper panels). The ethidium bromide-stained gels used for northern blot analysis served as a loading control (rRNA is indicated). Polysome profiling analysis of *L. infantum* promastigotes untreated (no stress) or treated with 0.4 µM of thapsigargin (Tg) for 4 hours (B, lower panels). Data shown here are representative of at least four independent experiments with similar results.

Under promastigote growth conditions (25°C, pH 7.3), the *L. infantum* alpha-tubulin transcript was predominantly associated with highly translating polysomes (Figure 5A). However, both heat stress and to a larger extent the combined temperature and low pH stress promoted a shift of the alpha-tubulin mRNA from the highly translating polysomes to the monosomes and ribosome-free fractions (Figure 5A), consistent with reduced translation under these conditions (Figure 1, bottom panels). Similarly, attenuation of general translation following endoplasmic reticulum (ER) stress induced by pharmacological agents such as thapsigargin (Figure 5B, bottom panel) or tunicamycin (data not shown) had an effect on the efficiency of alpha-tubulin mRNA translation. Indeed, increasing concentrations of thapsigargin or tunicamycin treatment resulted in gradual dissociation of the alpha-tubulin mRNA from the polyribosomes (Figure 5B, upper panels). The decrease in translation observed upon ER stress was not due to parasite mortality, as *Leishmania* treated with 1.0 µM thapsigargin for 4 hrs exhibited only 1% mortality in comparison to the untreated control (data not shown). Together these findings indicate that upon various stresses leading to a reduction in global translation, housekeeping transcripts such as alpha-tubulin are less efficiently translated.

**Figure 6:**
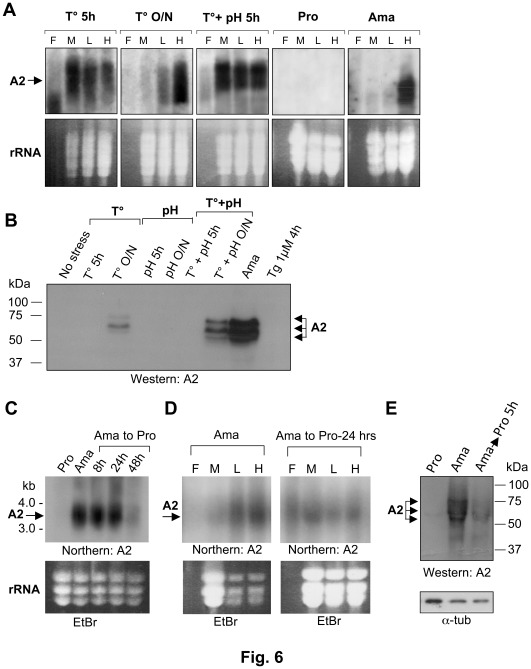
Translation of *L. infantum* developmentally regulated transcripts under temperature and acidic pH stresses. (A) Northern blot hybridization of *L. infantum* RNA isolated from sucrose gradient fractions (F, M, L and H are as in Figure 5) to assess the association of the amastigote-specific *A2* transcript with the ribosomes under temperature stress or acidic pH stress or a combination of both stresses. The ethidium bromide-stained gels used for northern blot analysis served as a loading control (rRNA is indicated). (B) Western blot analysis of *L. infantum* protein lysates using a monoclonal anti-A2 antibody to determine expression levels of A2 protein under different stresses, including high temperature, low pH and Tg-induced ER stresses. Controls using *L. infantum* promastigotes (Pro) (non stress) or axenic amastigotes (Ama) were included for both northern and western blots. (C) Northern blot hybridization of *L. infantum* total RNA to determine *A2* mRNA expression during amastigote to promastigote differentiation for up to 48 hrs. (D) Northern blot hybridization of *L. infantum* RNA isolated from sucrose gradient fractions to determine the association of the *A2* mRNA with monosomes or translating polysomes. (E) Western blot with an anti-A2 antibody of *L. infantum* amastigotes transferred into MAA-20 medium at 25°C and pH 7.3 for up to 5 hours to allow its differentiation into promastigote forms. The anti-alpha-tubulin antibody was used as loading control. Data shown here are representative of three independent experiments with similar results.

In contrast to the alpha-tubulin mRNA, translation of the stage-regulated *A2* transcript markedly increased under heat stress. The *A2* transcript is specifically expressed in amastigotes (Figure 6A and 6C and [41]), but elevated temperature and pH stress can also trigger its expression [10]. Northern blot hybridization revealed that 5 hours following of *L. infantum* exposure to elevated temperature or to a combined temperature and acidic pH stress, the *A2* transcript was detected both in the monosome and polysome fractions (Figure 6A). Its partial association with the polysomes did not result however, in high levels of translated A2 protein as determined by western blot with a specific anti-A2 antibody (Figure 6B). However, after an O/N exposure of the parasite to heat stress, the *A2* transcript shifted mainly to the highly translating polysome fraction, suggesting an active translation (Figure 6A) as also confirmed by western blot analysis (Figure 6B). In contrast to heat stress, acidic pH stress alone did not trigger A2 expression (Figure 6B). In addition, thapsigargin-induced ER stress, shown here to decrease global translation in *L. infantum* (Figure 5B, lower panel), did not induce *A2* mRNA accumulation (data not shown) or protein expression (Figure 6B). In axenic amastigotes, the totality of *A2* mRNA was found associated with the heavy polysomes (Figure 6A) leading to high levels of translation as confirmed by western blot analysis (Figure 6B). Interestingly, reconversion of amastigotes back to promastigote growth conditions resulted in a marked decrease in *A2* mRNA accumulation (Figure 6C), a shift of the *A2* mRNA association from translating polyribosomes towards the monosome and free-ribosome fraction (Figure 6D) and a significant reduction in A2 protein synthesis (Figure 6E). Although no significant decrease in the accumulation of *A2* mRNA was observed during the first 24 hrs following the amastigote to promastigote switch (Figure 6C), western blot analysis revealed lower A2 protein synthesis already 5 hours after the switch (Figure 6E). Altogether, these findings suggest that A2 developmental regulation occurs mainly at the translational level.

## Discussion

In this study, we explored the role of translational control in the context of *Leishmania* differentiation from free-living promastigotes to intracellular amastigote forms and vice-versa. We provide evidence that protein synthesis is generally reduced under growth conditions inducing amastigote differentiation and that reduced translation rates coincide with the phosphorylation of the alpha-subunit of eukaryotic initiation factor-2. Interestingly, we show that attenuation of global translation is not only observed in parasites transiently subjected to the amastigote differentiation signal (e.g. elevated temperature and acidic pH), but also in adapted amastigote cultures, suggesting that this life stage has to enter a slow growth state in order to adapt to the harsh environment in the phagolysosomal compartment. In addition, we show that while general translation is decreased during amastigote differentiation, translation of developmentally regulated transcripts like *A2* is preferentially enhanced, in agreement with the induction of an amastigote-specific differentiation program.

During its amastigote differentiation inside macrophages, *Leishmania* experiences an important number of stressful conditions, including rise in temperature, a dramatic shift in extracellular pH, increased levels of oxygen and nitrogen-reactive species, a high proteolytic activity and a nutritional stress within the phagolysosome. These external stimuli induce dynamic alterations in the regulation of gene expression, mainly at the posttranscriptional level [6], [7], leading to important morphological and biochemical changes that ensure the parasite’s intracellular survival. It has been established previously that temperature shift (from 25°C to 37°C) and drop in pH (from pH 7.3 to pH 5.5) provide a key signal for promastigote to amastigote differentiation [8], [10]. Here, we show that exposure of *L. infantum* promastigotes to a combined elevated temperature and acidic pH stress for few hours (3–5 hrs) leads to a marked decrease in general translation. A similar decrease in polysome formation following the differentiation signal has been reported recently in *L. donovani* but after longer exposure times (e.g. 15–24 hrs) [8], [9], [10], [11]. Of most interest, we show that translation remains generally lower in adapted amastigotes grown for several passages in a cell-free medium. Reconversion of amastigotes back to promastigote growth conditions results in increased levels of general translation comparable to those of promastigotes, suggesting that translational control plays a key role during the *Leishmania’*s development. Thus, the highly replicating promastigote forms undergo more active translation than intracellular amastigotes. These conclusions are consistent with previous reports demonstrating preferential upregulation in promastigotes of genes involved in translation (e.g. ribosomal proteins, translation factors, tRNA synthetases) [13], [14].

Eukaryotic cells have evolved mechanisms to respond to various cellular stresses encountered in their environments. One of the best-characterized stress response pathways conserved from yeast to humans is the reversible phosphorylation of the alpha-subunit of eukaryotic initiation factor 2, which lowers global protein synthesis along with induced translation of selected mRNAs [23], [25]. Here, we show that attenuation of general translation upon environmental stimuli such as elevated temperature and acidic pH triggering promastigote to amastigote differentiation or during amastigote growth is associated with eIF2α phosphorylation. These data suggest that *Leishmania* infective promastigotes experience stress upon their entry in macrophages and that translation attenuation mediated by eIF2α phosphorylation is important to allow the necessary adaptations for the parasite’s intracellular survival in the hostile environment of the phagolysosome. EIF2α phosphorylation is linked to amastigote differentiation as reconversion of amastigotes back to promastigote forms results in a dramatic decrease of eIF2α phosphorylation levels and upregulation of translation. Recent work from our laboratory highlighted the importance of eIF2α phosphorylation for amastigote differentiation as parasites unable to phosphorylate eIF2α by the ER resident PERK eIF2α kinase were markedly delayed in their differentiation into amastigote forms within macrophages [27]. The importance of eIF2α phosphorylation in the intracellular development of parasitic protozoa has been emphasized not only in *Trypanosomatidae* such as *Leishmania* [27] and *Trypanosoma cruzi* [42], but also in *Apicomplexa* [27], [37], [43], [44]. For example, in *Toxoplasma gondii,* it has been shown that eIF2α phosphorylation is important for maintaining latency of the bradyzoite form in the mammalian host [44], [45]. Moreover, it has been reported that phosphorylation of the *Toxoplasma* eIF2α is critical for the resistance of the parasite to external stress when outside its host and that inhibition of this process significantly delays the development of acute toxoplasmosis *in vivo* [44]. In *Plasmodium*, phosphorylation of PfeIF2α by the IK2 kinase plays an important role in salivary gland sporozoite latency and transformation into liver stages [37]. While combined heat stress (37°C, a physiological temperature encountered within macrophages) and drop in pH promote a reduction in global translation and eIF2α phosphorylation in *Leishmania*, interestingly in the related parasite *Trypanosoma brucei,* it has been shown that heat-shock stress up to 41°C causes a decrease in polysomes and the formation of stress granules but independently of eIF2α phosphorylation [31]. It is possible that under extreme stress conditions such as heat-shock at 41°C, mechanisms other than eIF2α phosphorylation may operate in these parasites to reduce levels of translation.

Reduced translation during amastigote differentiation may allow *Leishmania* to conserve energy while reconfigurating the expression of specific sets of genes necessary for its survival in the mammalian host. Consistent with this hypothesis it has been shown previously that the effects of elevated temperature and/or acidic pH induce the expression of several individual mRNAs [13], [14], [17], [30]. Here we show that although translation of housekeeping genes like alpha-tubulin is reduced during amastigote differentiation, translation of developmentally regulated transcripts is selectively upregulated. This was the case of the *A2* amastigote-specific gene [41] whose translation markedly increased upon exposure to elevated temperature or to the differentiation signal. Our findings support that developmental regulation of the *A2* transcript occurs mainly at the level of translation and that is triggered by elevated temperature and not by drop in the pH. Through its higher association with polyribosomes, the *A2* transcript is stabilized and is more efficiently translated. When the temperature shifts from 37°C to 25°C during amastigote to promastigote differentiation, the *A2* transcript is not longer associated with translating polyribosomes and is being gradually degraded. Our data indicate that although the *A2* transcript could be detected for more than 24 hrs following amastigote to promastigote differentiation *in vitro*, A2 translation was significantly diminished already ∼5 hrs upon differentiation, suggesting that A2 downregulation during amastigote to promastigote switch is also controlled at the level of translation. It has been shown recently that the A2 protein is induced by heat shock (40°C) and that under these conditions is complexed with the ER chaperone BiP [46], suggesting that A2 is a stress response protein that may play a role in enabling *L. donovani* to survive the higher temperatures associated with visceral organ infection. Despite its suggested localization in the ER following heat stress, our data indicate that A2 protein expression is not induced upon ER stress.

In this study, the findings indicate that *Leishmania* is capable of withstanding stressful situations during its digenetic life cycle by regulating general translation through reversible eIF2α phosphorylation. Global attenuation of translation during amastigote differentiation likely explains the slower growth rate of amastigotes inside the phagolysosome under conditions where metabolic requirements are reduced as compared to extracellular promastigotes [47]. Translational control seems also to be key in the regulation of amastigote-specific genes such as the *A2* gene. Overall, these findings contribute to our better understanding of the adaptive responses of *Leishmania* to stress during its developmental switches and highlight the importance of translational control in promastigote to amastigote differentiation and vice-versa.

## Materials and Methods

### Cell Culture

The *Leishmania infantum* MHOM/MA/67/ITMAP-263 strain used in this study has been described elsewhere [32]. Promastigotes were cultured at 25°C and pH 7.3 in RPMI-1640 medium supplemented with 1 µg/ml d-biotin, 20 µg/ml adenosine, 5 µg/ml hemin and 10% heat-inactivated fetal calf serum (Multicell, Wisent Inc). Acidic stress was induced in RPMI medium at pH 5.5. *L. infantum* promastigote to amastigote differentiation in a cell-free culture and the maintenance of axenic amastigotes were carried out as described previously [9], [48]. Typically, late stationary-phase promastigotes were inoculated in MAA/20 medium supplemented with 20% serum in 25-cm^2^ ventilated flasks and grown at 37°C and pH 5.5 with 5% CO_2_ for 5 days in average.

### RNA and Protein Manipulations

Total RNA was isolated with Trizol™ (Invitrogen) following the manufacturer’s instructions. Northern blot hybridizations were performed following standard procedures [49]. *Leishmania* lysates were clarified by centrifugation at 13,000×*g* for 15 min at 4^o^ C. Protein quantification was assessed using the Bradford reagent (BioRad) and 25 µg of total protein lysates was loaded onto 10% SDS-PAGE. The gels were transferred on Immobilon-P polyvinylidene difluoride membranes (Millipore) following the manufacturer’s instructions. For the anti-alpha tubulin and anti-A2 antibodies, blocking was carried out for 60 min in phosphate-buffered saline with 5% non fat dry skim milk or 5% BSA, respectively prior to the addition of the first antibody. The A2 antibody was kindly provided by Dr Greg Matlashewski (McGill University). For the anti-eIF2alpha [pS51] phosphospecific antibody, blocking was performed O/N at 4°C in 5% BSA, prior to incubation for 2 hrs at RT° in 1% BSA. Mouse monoclonal antisera raised against *L. infantum* eIF2alpha [27], mouse monoclonal anti-alpha tubulin antibody (Sigma) and rabbit polyclonal anti-eIF2alpha (human) [pS51] phosphospecific antibody (BioSource™) were used at 1∶1000 dilutions. Phosphate-buffered saline with 0.1% Tween 20 was used to wash the membranes after the blocking step. Anti-mouse or anti-rabbit antibodies that had been conjugated to horseradish peroxidase (Santa Cruz Biotechnology) were diluted at 1∶10,000 in PBS-Tween 0.1% and 5% non fat dry skim milk and added to the membrane for 60 min. Three X 10 min washes (PBS with 0.1% Tween 20) were performed before visualizing the signal on the membrane by chemiluminescence using the Amersham Hyperfilm™/ECL™ kit (GE HealthCare). After exposure of the film to the membrane, protein amounts were quantified by densitometric analyses using a PhosphorImager with ImageQuaNT 3.1 software.

### Polysome Profiling Analysis

A total of 3×10^9^ *L. infantum* late-log/stationary-phase promastigotes treated with 0.4 µM or 1 µM thapsigargin or 5 µg/ml tunicamycin (Sigma) or subjected to heat-shock or acidic stress or to amastigote differentiation in culture were incubated with 100 µg/ml cycloheximide (Sigma) for 10 min, washed with cycloheximide-containing PBS buffer and lysed with a Dounce homogenizer in lysis buffer [10 mM Tris-HCl pH 7.4, 150 mM NaCl, 10 mM MgCl_2_, 1 mM DTT, 0.5% IGEPAL, 100 µg/ml cycloheximide, 100 U/ml RNAseOUT (Amersham), 1 mM PMSF, 15 µl/ml of protease inhibitor cocktail (Sigma)]. *Leishmania* lysates were pelleted by centrifugation and the supernatant (40 OD_260 nm_ units) was layered on top of a 15% to 45% linear sucrose gradient (10 ml) in gradient buffer (50 mM Tris-HCl pH 7.4, 50 mM KCl, 10 mM MgCl_2_, 1 mM DTT, 100 U/ml RNaseOUT) as described previously [27], [50]. Ribosomal subunits (40S and 60S), monosomes (80S) and polyribosomes were sedimented by centrifugation in a Beckman SW40 Ti rotor at 35,000 rpm for 2.15 hours at 4°C and fractions were collected using an ISCO Density Gradient Fractionation System under constant monitoring of the absorbance at 254 nm. RNA was extracted from the free subunit (F), monosome (M), light polysome (L) and heavy polysome (H) fractions by phenol-chloroform followed by ethanol precipitation and analyzed by northern blot hybridization.

### Metabolic Labeling

Approximately 5×10^7^ *L. infantum* promastigotes grown in RPMI-1640 medium were subjected to either heat-shock from 25°C to 37°C or to low pH (from pH 7. 3 to pH 5.5) or to both conditions for different time periods. Thirty minutes before each time point, 0.5 ml of the culture was transferred to a methionine-free medium (Gibco) supplemented with 1 µCi/ml [^35^S]-methionine (GE Healthcare) and re-incubated under the same conditions for the rest of the stress period. Cells were pelleted, washed twice with ice-cold phosphate-buffer saline (PBS) and lysed in SDS-PAGE sample buffer. [^35^S]-methionine incorporation was measured with a scintillation counter (Beckman LS 6000TA). Samples were analyzed by SDS-PAGE and autoradiography.

### Pulse Chase Assay

*L. infantum* promastigotes were washed twice in PBS and incubated in a methionine-free medium supplemented with 1 µCi/ml [^35^S]-methionine for 1 hour at 25°C. Following two washes in PBS, stress was induced in a cold methionine-containing medium (Gibco) at 37°C and pH 5.5. After different time points, 0.5 ml of culture were washed twice in PBS and the cells were lysed in Laemmli buffer. Non stressed cells were grown for the same period as the stressed cells and treated the same way. Values were normalized with the non-stressed control. [^35^S]-methionine incorporation was measured with a scintillation counter (Beckman LS 6000TA).

## Supporting Information

Figure S1 **Global translation is reduced in** ***L. major*** **promastigotes subjected to temperature and acidic pH stress.** Polysome profile analysis of *L. major* LV39 promastigotes (no stress) and parasites exposed to a combined stress of elevated temperature (37°C) and acidic pH (5.5) for 4 hours. Cell lysates were sedimented on 15% to 45% sucrose gradients. Gradients were fractionated and absorbance (Abs) at 254 nm was continuously recorded. The 40S and 60S subunits, 80S monosome and polysome peaks are indicated. Data displayed represent one of two separate experiments.
(TIF)

Figure S2 **Morphological changes of** ***L. infantum*** **during axenic amastigote differentiation.** Morphological analysis of *L. infantum* axenic differentiation from elongated flagellated promastigotes into round aflagellated amastigote-like forms using a phase-contrast microscope. *L. infantum* promastigotes (Pro) exposed to either elevated temperature (37°C) or a combination of high temperature and low pH (pH 5.5) for 4 and 8 hours, respectively are shown here.
(TIF)
