# Supplementary figures and images for: Translational Control through eIF2alpha Phosphorylation during the Leishmania Differentiation Process
Source: PLoS One. 2012 May 31;7(5):e35085. doi: 10.1371/journal.pone.0035085 (PMC3365078; doi:10.1371/journal.pone.0035085)

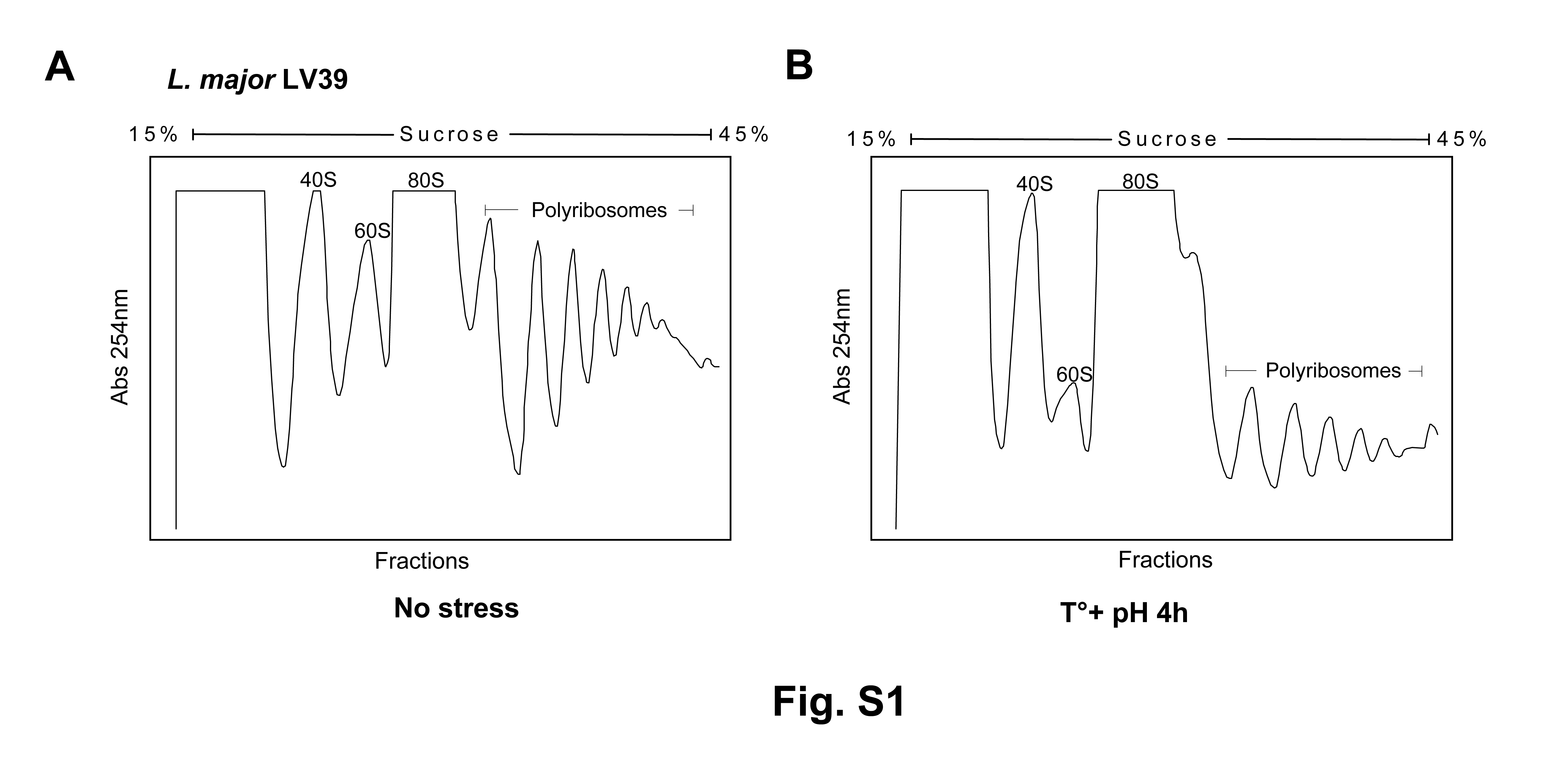

Supplement: Figure S1 — Global translation is reduced in L. major promastigotes subjected to temperature and acidic pH stress. Polysome profile analysis of L. major LV39 promastigotes (no stress) and parasites exposed to a combined stress of elevated temperature (37°C) and acidic pH (5.5) for 4 hours. Cell lysates were sedimented on 15% to 45% sucrose gradients. Gradients were fractionated and absorbance (Abs) at 254 nm was continuously recorded. The 40S and 60S subunits, 80S monosome and polysome peaks are indicated. Data displayed represent one of two separate experiments. (TIF) [file pone.0035085.s001.tif]

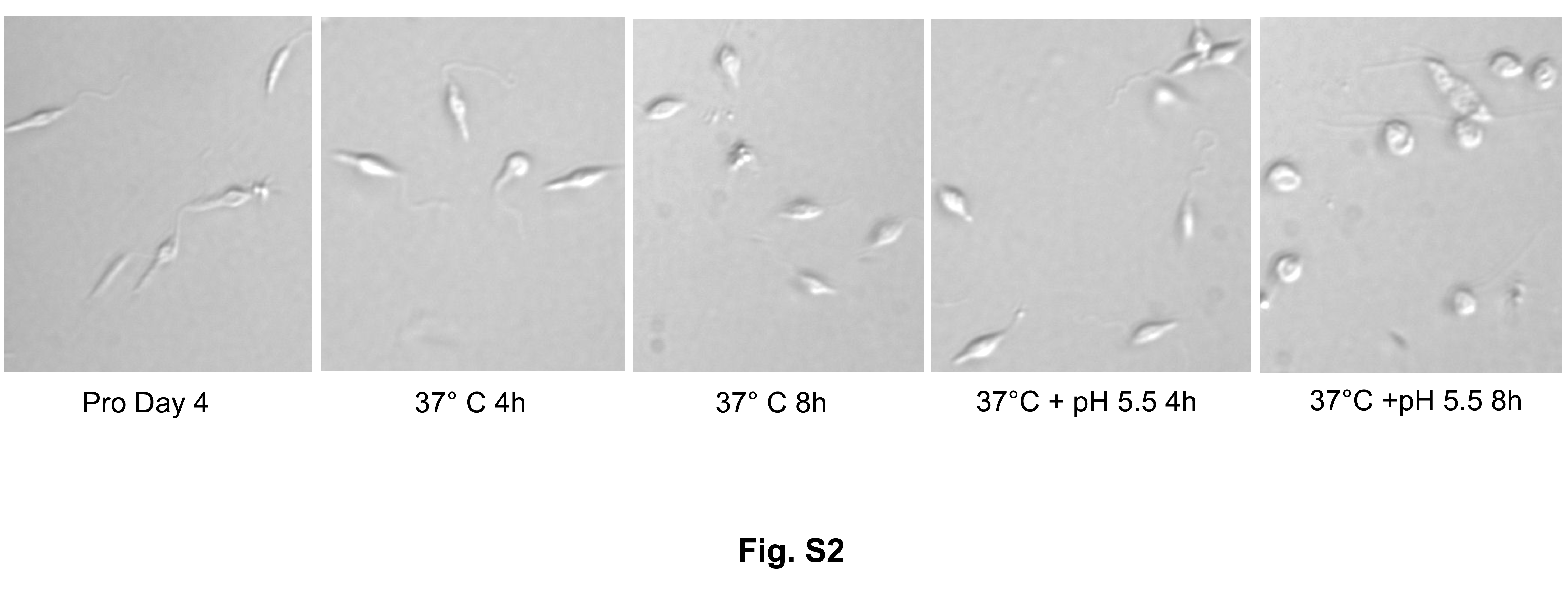

Supplement: Figure S2 — Morphological changes of L. infantum during axenic amastigote differentiation. Morphological analysis of L. infantum axenic differentiation from elongated flagellated promastigotes into round aflagellated amastigote-like forms using a phase-contrast microscope. L. infantum promastigotes (Pro) exposed to either elevated temperature (37°C) or a combination of high temperature and low pH (pH 5.5) for 4 and 8 hours, respectively are shown here. (TIF) [file pone.0035085.s002.tif]
